# Survival Rate of Prostate Cancer in Asian Countries: A Systematic Review and Meta-Analysis

**DOI:** 10.5334/aogh.2607

**Published:** 2020-01-02

**Authors:** Soheil Hassanipour, Hamed Delam, Morteza Arab-Zozani, Elham Abdzadeh, Seyyed Ali Hosseini, Hossein-Ali Nikbakht, Mahdi Malakoutikhah, Mohammad Taghi Ashoobi, Mohammad Fathalipour, Hamid Salehiniya, Shirin Riahi

**Affiliations:** 1 Gastrointestinal and Liver Diseases Research Center, Guilan University of Medical Sciences, Rasht, IR; 2 GI Cancer Screening and Prevention Research Center, Guilan University of Medical Sciences, Rasht, IR; 3 Student Research Committee, Larestan University of Medical Sciences, Larestan, IR; 4 Social Determinants of Health Research Center, Birjand University of Medical Sciences, Birjand, IR; 5 Iranian Center of Excellence in Health Management, School of Management and Medical Informatics, Tabriz University of Medical Sciences, Tabriz, IR; 6 English Department, Paramedical School, Shiraz University of Medical Sciences, Shiraz, IR; 7 Social Determinants of Health Research Center, Health Research Institute, Babol University of Medical Sciences, Babol, IR; 8 Department of Occupational Health, Kashan University of Medical Sciences, Kashan, IR; 9 Department of Pharmacology, Faculty of Pharmacy, Hormozgan University of Medical Sciences, Bandar Abbas, IR; 10 Non-communicable Diseases Research Center, Alborz University of Medical Sciences, Karaj, IR

## Abstract

**Background:**

Prostate cancer is one of the most common health issues among men, especially older men. In recent years, incidences of prostate cancer is increasing.

**Objective:**

The aim of this study was to provide a comprehensive estimate of the survival of prostate cancer in Asian countries.

**Methods:**

We searched five international databases including Medline/PubMed, Scopus, Embase, Web of Knowledge and ProQuest until June 1, 2018. The Newcastle-Ottawa Quality Assessment was used to evaluate the quality of selected papers. The review protocol was registered in PROSPERO (CRD42019117044).

**Results:**

A total of 714 titles were retrieved. Thirty-seven studies met the inclusion criteria. Based on the random-effect model one-year, five-year and ten-year survival rate of prostate cancer were 81% (95% CI 77.8–84.2), 61.9% (95% CI 59.5–64.3) and 36.2% (95% CI 9.2–63.2) respectively. Survival rates based on HDI level for five-year were 30.07, 43.43 and 70.84 percent for medium, high and very high levels, respectively.

**Conclusion:**

According to the results of our study, the prostate cancer survival rate in Asian countries is relatively lower than in Europe and North America.

## Introduction

Prostate cancer, the sixth most common cancer of all cancers, is the second most frequent malignancy in men around the world as well as the most common cancer in men in Europe, North America and parts of Africa [1,2]. The number of new cases in 2000 amounted to 513,000 people, in 2008 nearly 900,000 people (33 per 100,000 population) and in 2012, about 1.1 million cases were estimated; it is expected that in 2030, the number of new cases of the disease will reach about 1.7 million people and the number of deaths is estimated to extend to 499,000 people globally [3,4,5]. Recent international studies on the epidemiology of prostate cancer indicate a high incidence of this disease in Western countries [2,6]. The highest incidences of this cancer are observed in Australia/New Zealand, North America, and northern and western parts of Europe [7,8,9]. On the other hand, over the past few decades, the incidence of prostate cancer in Asian countries has been much lower [7,10,11]. However, significant economic growth as well as social and cultural adjustments in some Asian countries have led to an increase in life expectancy, which has ultimately expanded the incidence and mortality of these cancers in these countries [12,13]. The average mortality rate from this cancer in Asian countries was 3.8 per 100,000 [14]. The low incidence of prostate cancer in Asian countries can be due to various reasons, such as the lack of access to screening tests for diagnosis, other features including nutritional status, genetics, lifestyle, environmental factors, physical activity, smoking, race and registration cancer system [10,15,16]. Survival rates are one of the most important indicators for assessing the quality of cancer control and treatment programs. According to studies, the survival rate of patients with cancer has increased in recent years [17]. Several studies which are conducted on the survival of this cancer in Asia have reported various results, as an illustration, see Chen et al. In the period of 1992 to 2000 in China, a five-year relative survival rate of prostate cancer estimated about 32.5% [18]. However, Jung et al.’s study in South Korea for the two periods of 1996 and 2010–2014 revealed 67.2% and 93.3% survival rate respectively [18,19,20,21]. In a study in Iran, the overall five-year survival rate was 36.1% [22]. In another study conducted among different ethnic groups in China, the survival rate was reported to be 26.6% to 78% over the years, which showed a noticeable fluctuation trend and there was a significant difference between various ethnic groups [23]. In another study by Xu et al. in China, there was a significant difference between the five-year survival of prostate cancer patients with hypertension (28.5%) and the control population (48.3%) [24].

Accurate information on the survival rate of prostate cancer is essential, as it is used in various health and diagnostic planning [25,26]. There is no comprehensive estimate of the survival rate of prostate cancer in Asian countries. Considering the importance of knowledge of the survival rate of this cancer in hospital planning and the different results between published articles, the purpose of this study was to provide a comprehensive estimate of the survival of prostate cancer patients in Asian countries through systematic review and meta-analysis.

## Material and methods

The present study is a systematic review and meta-analysis study of prostate cancer survival in Asian countries. This study was designed and conducted in 2018. The methodology of the present study is based on the PRISMA (Preferred Reporting Items for Systematic Reviews and Meta-Analyses) statement [27]. The review protocol was registered in PROSPERO (CRD42019117044).

### Search strategy

The researchers searched five international databases including Medline/PubMed, Scopus, Embase, Web of Knowledge, and ProQuest until June 1, 2018. We also searched the Google Scholar for detecting grey literature. Selected keywords for international databases included: (“Neoplasm”, “Cancer”, “Carcinoma”, “Malignancy”, “prostate Cancer”, “prostate Neoplasms”, “prostate carcinoma”, “prostate Tumor”, “Cancer of prostate”, “Neoplasms of prostate”, “Survival”, “Survival Analysis”, “Survival Rate”, “Afghanistan”, “Armenia”, “Azerbaijan”, “Bahrain”, “Bangladesh”, “Bhutan”, “Brunei”, “Myanmar”, “Cambodia”, “China”, “Georgia”, “Hong Kong”, “India”, “Indonesia”, “Iran”, “Iraq”, “Israel”, “Japan”, “Jordan”, “Kazakhstan”, “North Korea”, “South Korea”, “Kuwait”, “Kyrgyzstan”, “Laos”, “Lebanon”, “Macau”, “Malaysia”, “Maldives”, “Mongolia”, “Nepal”, “Oman”, “Pakistan”, “Philippines”, “Qatar”, “Saudi Arabia”, “Singapore”, “Sri Lanka”, “Syria”, “Taiwan”, “Tajikistan”, “Thailand”, “Timor-Leste”, “Turkmenistan”, “United Arab Emirates”, “Uzbekistan”, “Vietnam”, and “Yemen”).

The initial search was conducted by two researchers (SR and MF). The searched record entered the EndNote X7 software, and duplicate articles were deleted.

### Inclusion and exclusion criteria

All observational studies (cross-sectional, case-control, and cohort) stated the survival rate of localized prostate cancer in Asian countries were included in the study. Articles of other cancers reported survival in people who reported regional, metastatic, as well as review and meta-analysis studies were excluded. It should be noted that studies that did not report the sample size or confidence interval of survival rates were not included in the meta-analysis.

### Quality assessment

The Newcastle-Ottawa Quality Assessment Form was used to evaluate the quality of selected papers. This tool has three different parts including selection (4 questions), comparability (1 question) and outcome (3 questions) and based on the final scores divided into three categories: good (3 or 4 stars in selection domain and 1 or 2 stars in comparability domain and 2 or 3 stars in outcome/exposure domain), fair (2 stars in selection domain and 1 or 2 stars in comparability domain and 2 or 3 stars in outcome/exposure domain) and poor (0 or 1 star in selection domain or 0 stars in comparability domain or 0 or 1 stars in outcome/exposure domain) [28]. Results of quality assessment are presented in Appendix 1.

### Screening of studies

Screening of studies, extraction of results, and evaluation of quality control of articles were performed separately independently by two authors (HD and EA). If there was no agreement between the two, the supervisor (SH) would announce the final comment on that article.

### Data extraction form

All final articles entered into the study process were provided by a checklist that was previously prepared, and were arranged to extract the data. This checklist includes the name of the author, the year of publication, the period of the study, the country of origin, the survival rate by year for each survival period.

### Statistical analysis

The heterogeneity of the studies was assessed by Cochran test (with significance less than 0.1) and its composition using I^2^ statistics. In the case of heterogeneity, the random-effects model was utilized with the inverse-variance method, and in the absence of heterogeneity, the fixed-effects model was applied. In the case of a heterogeneity in the studies, methods such as subgroup analysis were used and factors like the geographical area and the HDI considered in the analysis of subgroups. All analyzes were performed by the STATA (version 13) software.

### Additional analysis

Due to the heterogeneity of the studies, the subgroups analysis was used. The indicator applied for this purpose is Human Development Index (HDI). The HDI is a relative measure of life expectancy, education, quality and education level, and in general, it is the living standard in human societies. This index is estimated using the measure of welfare, especially among children and people of low age. These statistics can be used to measure the development of countries, the impact of economic policies on living standards, and the survival of prostate cancer in each of the countries was reported to provide a clear indication of the prostate cancer survival status in each country [29].

## Results

### Study selection

**Figure 1:**
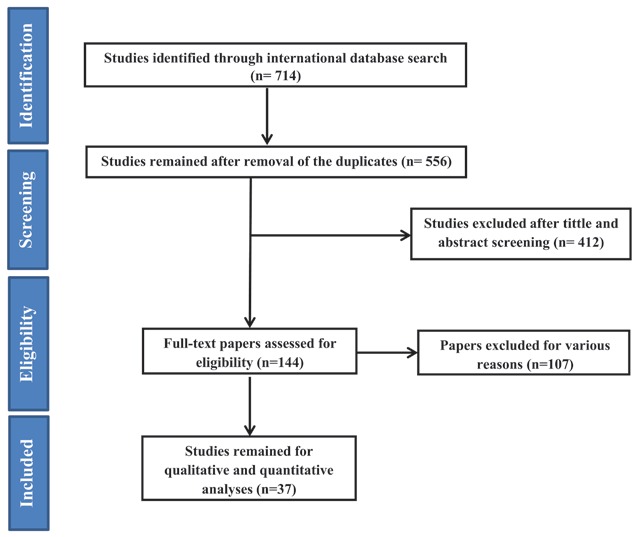
Flowchart of the included eligible studies in systematic review.

After searching all the international databases, 714 articles were selected and after removing duplicate articles, the total was 556. After reviewing the titles and abstract of articles, 144 articles entered the next stage, at which point the full text was examined and 37 articles entered the final analysis. It should be noted that the referenced articles were also reviewed to add related studies. In the screening stages, some articles were excluded for a variety of reasons, which included unrelated topic (N = 289), unrelated population (N = 182), inadequate information (N = 44) and repeated results (N = 4). The study selection process is outlined in Figure 1.

### Study characteristics

The included studies were published from 1998 to 2018. Based on geographical locations, nine studies were conducted in Korea [19,20,21,30,31,32,33,34,35], five in China [18,36,37,38,39], five in Japan [40,41,42,43,44], four in India [45,46,47,48], four in Thailand [49,50,51,52], two in Singapore [53,54], two in Taiwan [55,56], one in Hong Kong [57], one in the Philippines [58], one involved Chinese residents in Singapore [59], one involved countries (China, India and Singapore) [60], one involved the Asian population in England [61]. Characteristics of the included studies are presented in Table 1.

**Table 1:** Basic information of included studies.

Order	Author (year)	Location	Time period	Sample size	Survival Rate		

					1	5	10

1	Esteban (1998)	Philippines	1987	58	66.00	15.60	NR
2	Fan Jin (1998)	China	1988–1991	373	62.50	29.60	NR
3	Martin (1998)	Thailand	1983–1992	157	65.20	28.70	NR
4	Sato (2002)	Japan	1962–1966	27	NR	36.30	NR
			1967–1971	32	NR	19.4	NR
			1972–1976	46	NR	37.6	NR
			1977–1981	51	NR	31.3	NR
			1982–1986	58	NR	24.2	NR
			1987–1991	88	NR	73.4	NR
			1992–1994	114	NR	84.5	NR
5	Tsukuma (2006)	Japan	1993–1996	4348	88.6	50.2	NR
6	Jung (2007)	Korea	1993–1997	NR	NR	59.1	NR
			1998–2002		NR	70.6	NR
7	Lim (2009)	Singapore	1978–1982	NR	NR	NR	16.5
			1983–1987		NR	NR	30.1
			1988–1992		NR	NR	31.6
			1993–1997		NR	NR	41.6
			1998–2002		NR	NR	45.2
8	Chia (2010)	Chinese Residents in Singapore	1973–1977	NR	NR	51.3	NR
			1978–1982		NR	47.7	
			1983–1987		NR	55.7	
			1988–1992		NR	76.5	
			1993–1997		NR	76.3	
			1998–2002		NR	76.1	
9	Chen (2011)	China	1992–2000	55	37.7	15.2	NR
			1982–1991		NR	20.7	NR
10	Chia (2011)	Singapore	1993–1997	752	82.4	44.6	NR
11	Jayalekshmi (2011)	India	1991–1997	32	93.5	22.1	NR
12	Jung (2011)	Korea	1993–1995	NR	NR	55.9	NR
			1996–2000	NR	NR	67.2	NR
			2001–2005	NR	NR	79.5	NR
			2004–2008	NR	NR	86.2	NR
13	Law (2011)	Hong Kong	1996–2001	3206	87.8	55.6	NR
14	Martin (2011)	Thailand	1990–2000	171	80.7	53.1	NR
15	Matsuda (2011)	Japan	1993–1996	4220	NR	66.8	NR
			1997–1999	4508	NR	75.5	NR
			1993–1996	NR	NR	96.5	NR
			1997–1999	NR	NR	97.6	NR
16	Sankaranarayanan (2011)	China	1996–2001	NR	NR	78.2	NR
		China	1992–2000	NR	NR	36.3	NR
		China	1992–1995	NR	NR	54.2	NR
		China	1991–1999	NR	NR	68.4	NR
		India	1991–1997	NR	NR	32.6	NR
		India	1992–1999	NR	NR	42.3	NR
		Singapore	1993–1997	NR	NR	64.3	NR
17	Sriplung (2011)	Thailand	1990–1999	144	80.8	32.1	NR
18	Sumitsawan (2011)	Thailand	1993–1997	107	78.8	30.4	NR
19	Xiang (2011)	China	1992–1995	639	68.8	36.9	NR
20	Xishan (2011)	China	1991–1999	423	74.5	52.5	NR
			1981–1990	NR	NR	41.0	NR
21	Yeole (2011)	India	1992–1999	1463	64.3	24.0	NR
22	Chang (2012)	Taiwan	2002	5933	NR	77.1	NR
			2002	1392	NR	66.7	NR
			2002	1591	NR	69.3	NR
			2002	2761	NR	58.9	NR
23	Jung (2012)	Korea	1993–1995	NR	NR	55.9	NR
			1996–2000	NR	NR	67.2	NR
			2001–2005	NR	NR	79.9	NR
			2005–2009	NR	NR	87.6	NR
24	Balasubramaniam (2013)	India	1999–2002	371	92.0	62.0	NR
25	Ito (2013)	Japan	2000–2004	NR	81.0	87.0	NR
26	Jung (2013)	Korea	1993–1995	NR	NR	55.9	NR
			1996–2000	NR	NR	67.2	NR
			2001–2005	NR	NR	80.1	NR
			2006–2010	NR	NR	90.2	NR
27	Ito (2014)	Japan	2002–2006	19519	NR	89.2	78.0
			2001–2004	NR	94.2	79.6	NR
28	Jung (2014)	Korea	2007–2011	NR	NR	92.0	NR
29	Takiar (2014)	India	2011	1495	64.9	23.9	NR
30	Jung (2015)	Korea	2008–2012			92.3	NR
31	Maringe (2015)	England (South Asians)	1986–1995	310	92.3	54.4	NR
		England (Non-South Asians)	1986–1995	35550	84.4	51.0	NR
		England (South Asians)	1996–2004	1052	95.2	78.4	NR
		England (Non-South Asians)	1996–2004	72724	95.0	81.1	NR
32	Chang-Mo Oh (2016)	Korea	2008–2013	NR	NR	92.5	NR
33	Jung (2017)	Korea	2010–2014	NR	NR	93.3	NR
34	Chen (2018)	China	2002–2014	NR	69.05	18.80	9.40
			2002–2014	NR	NR	13.80	NR
			2002–2014	NR	NR	24.00	NR
35	Chien (2018)	Taiwan	2000–2010	NR	NR	80.06	NR
			2000–2010	NR	NR	71.62	NR
			2000–2010	NR	NR	76.6	NR
36	Jung (2018)	Korea	2011–2015	NR	NR	94.1	NR
37	Zeng (2018)	China	2003–2005	11690	NR	53·8	NR
			2006–2008		NR	60·8	NR
			2009–2011		NR	59·2	NR
			2012–2015		NR	66·4	NR

NR: Not Reported.

### Quality appraisal

The results of the quality assessment of the articles have been shown in Appendix 2. Based on our results, 35 studies have good and 2 studies have a fair quality.

### Results of the meta-analysis

First, the articles were sorted according to the year of publication and then analyzed by the one-, five- and ten-year survival rate. It should be noted that the number of papers discussing two- and four-year survival rate was very low, and the results of the three-year survival rate were not very reasonable due to that most of the less-developed countries and lower HDI were reported.

### One-year survival rate

**Figure 2:**
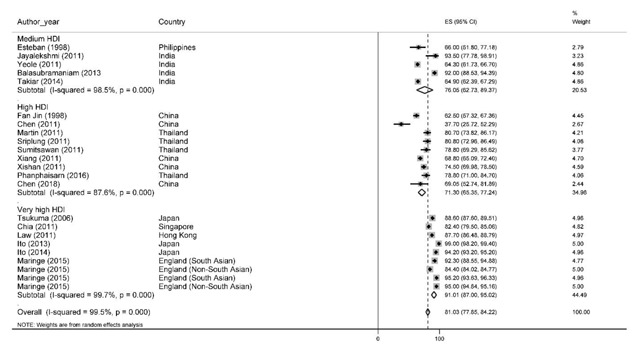
Forest plot of one-year survival rate of prostate cancer in Asian countries.

Of the most recent papers, 23 number reported one-year survival rate. Based on the random-effect model, the results of the study demonstrated that one-year survival rate in Asian countries was 81% with a 95% confidence interval of 77.8 to 84.2. One-year survival rate of prostate cancer based on HDI has been shown in Figure 2. According to the results, the highest one-year survival rate in countries with a high HDI level was 91% (95% CI, 95–87) and the lowest was for countries with high HDI levels 71.3% (95% CI, 77.3–65.3).

### Five-year survival rate

**Figure 3:**
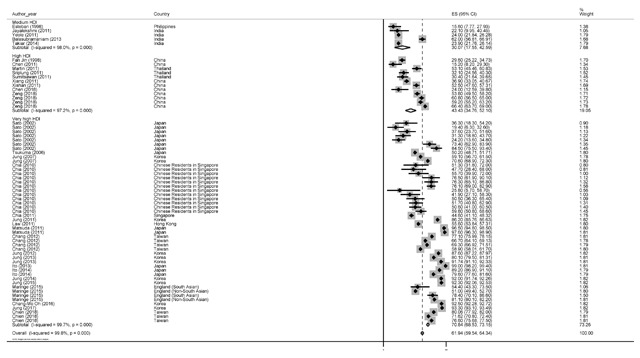
Forest plot of five-year survival rate of prostate cancer in Asian countries.

The five-year survival rate was 61.9% with a 95% confidence interval of 59.5 to 64.3. The results of the five-year survival rate by the HDI has been illustrated in figure Figure 3. Based on the findings of our study, the highest five-year survival rate for countries with a high HDI level was 70.8% (95% CI, 68.5–73.1) and the lowest among countries with a medium HDI level of 30% (95% CI, 17.5–42.5).

### Ten-year survival rate

**Figure 4:**
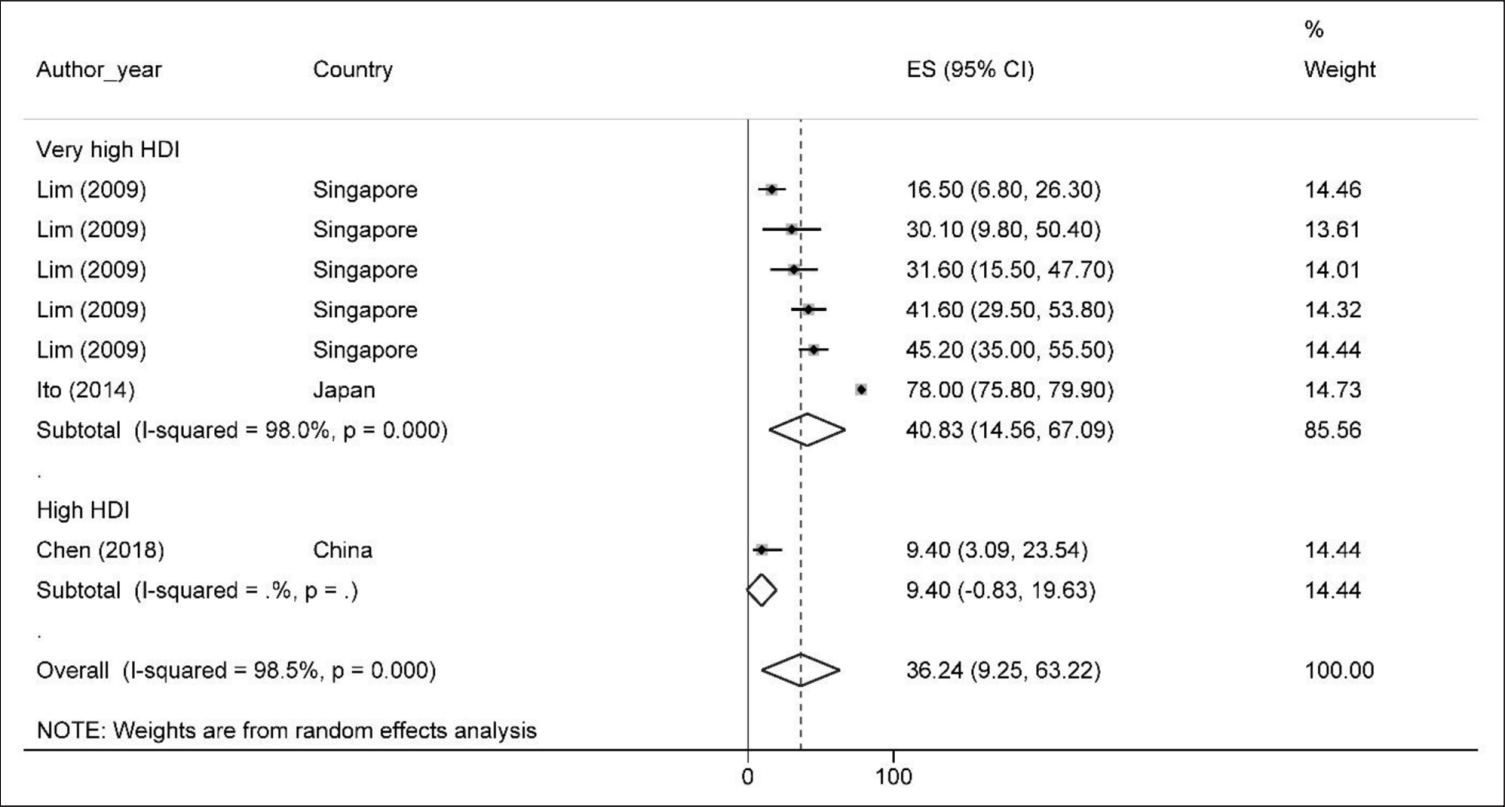
Forest plot of ten-year survival rate of prostate cancer in Asian countries.

A total of 11 studies reported 10-year survival rate. Based on the results, the 10-year survival rate was 36.2% with 95% confidence interval of 9.2 to 63.2. Ten-year survival rate of prostate cancer by HDI has been shown in Figure 4. Based on the results, the highest survival rates for countries with a high HDI level were 40.8% (95% CI, 14.5–67) and the lowest for countries with high HDI levels 9.4 (95% CI, 3–23.5). It should be noted that countries with medium HDI levels did not report the 10-year survival rate.

### Survival rate of prostate cancer in each country

Overall, the results of the survival of prostate cancer in nine countries and three other areas has been showed in the Table 2. The highest survival rates of one, five, and ten-years were reported in Japan (93), Korea (84.9), and Japan (78), respectively, and the lowest survival rates for these years were observed in China (64.1), Philippines (15.6), and China (9.4), respectively.

**Table 2:** Result of meta-analysis and heterogeneity of survival rate of prostate cancer in Asia based on each country and years of survival.

Country	Year of Survival											

	1				5				10			

	# of Study	Effect estimate	I^2^	P-value	# of Study	Effect estimate	I^2^	P-value	# of Study	Effectestimate	I^2^	P-value

China	5	64.1 (56.2–72)	88.1	<0.001	9	45 (35–54.9)	97.7	<0.001	1	9.4 (3–23.5)	–	–
Hong Kong	1	87.7 (86.5–88.8)	–	–	1	55.6 (53.8–57.3)	–	–	–	NR	NR	NR
India	4	78.2 (63.6–93.1)	98.9	<0.001	4	33.4 (19.3–47.5)	98.5	<0.001	–	NR	NR	NR
Japan	3	93.9 (87.6–99.8)	99.4	<0.001	13	64.2 (53.2–75.3)	99.7	<0.001	1	78 (75.9–80)	–	–
Philippines	1	66 (51.8–77.1)	–	–	1	15.6 (5.5–25.6)	–	–	–	NR	NR	NR
Singapore	1	82.4 (79.6–85.1)	–	–	1	44.6 (40.9–48.2)	–	–	5	33 (20.7–45.4)	78.3	<0.001
Korea	–	NR^*^	NR	NR	10	84.9 (82.8–97)	99.7	<0.001	–	NR	NR	NR
Taiwan	–	NR	NR	NR	7	71.5 (67.2–75.7)	98.6	<0.001	–	NR	NR	NR
Thailand	4	79.9 (76.4–83.3)	0	0.960	3	38.6 (23.9–53.3)	89.7	<0.001	–	NR	NR	NR
Chinese Residents in Singapore	–	NR	NR	NR	12	57.1 (48.9–65.3)	80	<0.001	–	NR	NR	NR
England (South Asian)	2	94.1 (91.3–96.8)	63.4	0.099	2	67.2 (43.8–90.7)	86.6	0.006	–	NR	NR	NR
England (Non-South Asian)	2	89.7 (79.3–99.6)	99.9	<0.001	2	66 (36.5–95.5)	99.9	<0.001	–	NR	NR	NR
Overall	24	81 (77.8–84.2)	99.5	<0.001	65	61.9 (59.5–64.3)	99.8	<0.001	7	36.2 (9.2–63.2)	98.5	<0.001

* NR: Not reported.

### Meta-regression

Results of meta-regression showed a significant association between publication year and five-year survival rate. Thus, year of study is a cause of variability in results of five-year survival rate (Reg Coef = 0.022, p < 0.001). This association for one-year survival rate not statistically significant (Reg Coef = –0.010, p = 0.084). According to results, an increasing survival rate across the study period was observed.

Another factor for inconsistency in results is HDI. HDI is a cause of variability in results of one (Reg Coef = 0.022, p < 0.001) and five-year survival rate (Reg Coef = 2.73, p < 0.001). According to results, an increasing survival rate was observed with higher HDI in countries.

**Figure 5:**
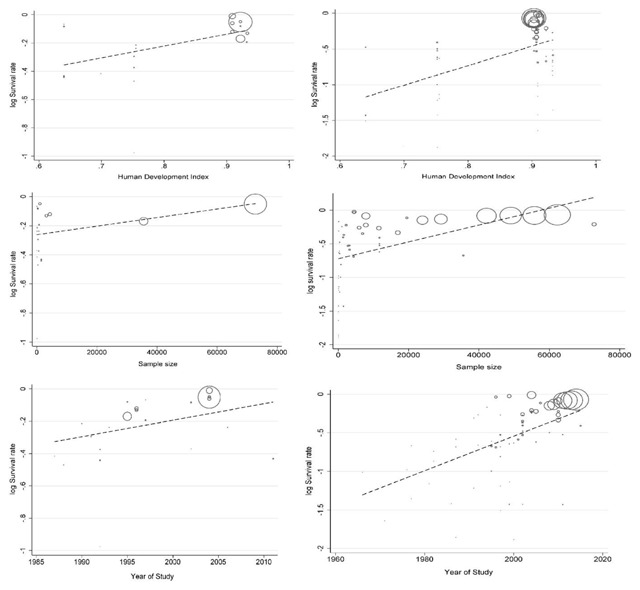
Result of meta-regression for one (left) and five-years (right) survival rate based on HDI, sample size and year of study.

Also, we examined the sample size as other explanatory factor to variability in our study and results showed sample size was another reason for this inconsistency (Reg Coef = –2.94 e^–6^, p = 0.194 for one-year, Coef = –0.00001, p = 0.001 for five-year survival rate). Studies with larger sample size had a higher survival rate, but this finding not statistically significant for one-year survival rate. Results of meta-regression is shown in Figure 5.

## Discussion

Prostate cancer is one of the most principal and arising health issues among elderly men, with a recent increase in its incidence[2]. Given that the importance of this cancer among men, especially in developing countries, as well as the many differences in the incidence and survival of this cancer in the world, awareness of survival is an important issue [62].

In this study, the survival rate of prostate cancer has been reported in Asia for one-, five- and ten-year survival rate. These values for the one-, five- and ten-year survival rate are 81.0 (77.8–84.2), 61.9 (59.5–64.3) and 36.2 (9.2–63.2) respectively.

One-year survival rates for countries with high HDI were lower than those with a medium HDI index. The reason for this difference is the low number of studies and those are the medium HDI often reported in India, while articles in the category of high HDI are mostly from China.

According to the results of this study, the highest survival rate has been reported in the Asians residence in England, followed by Japan in the second place. On the other hand, China has the lowest one-year survival rate. It seems that the higher HDI is directly related to the higher survival rate, as countries such as Japan and Singapore, which have a much higher HDI, have higher survival rates. But this is not true in India, because it has a higher survival rate, although it has a lower HDI than China. With regard to the three levels of the HDI, countries at the second level of this index have the lowest survival rates. However, given the low levels of countries at each level, especially in countries with a medium HDI, there can certainly not be any reason for this difference, and it seems to be due to variations in demographic characteristics [7]. Another potential reason can be observed in the short run of one-year, since studies have reveal that in longer periods of time, the survival rate is closer to real value [63].

This difference has not beeen seen in five-year survival rate and the aggregate survival rate for the three levels of HDI has a certain trend, so that countries with a medium HDI have the lowest survival rates and countries with a very high HDI have the highest aggregate survival rates. This higher survival rate could be better for reasons such as improving lifestyle, nutritional status, physical activity, and the use of health care [64].

According to the results of the Coleman et al. study, the five-year survival rate in 31 countries manifested that variations in the standardized survival of the age range are very wide between countries. This difference, even after adjusting for dissimilarities in mortality due to other causes, remains the highest in the United States, which was more inflated in Caucasians compared to African-Americans (92.4% versus 85.8%). These variations are due to reasons such as differences in treatment care and the stages of the diagnosed disease [65,66].

In another study by Steele et al., conducted in 2017, the results showed that the survival rates of one and five-years were significantly higher than the findings of the present study in the years 2002–2003 and 2004–2009. In the former study, the one-year survival rate for both periods was 98.6 and 98.8, while in the present study, this rate was 81%. The five-year survival rate was 96.7% and 96.9%, respectively, which was much higher than the results of the present study, which was 61.9% [67].

Critz et al. reported a 10-year survival rate of prostate cancer in their study in 2013 approximately 75% [68]. The results of this study, conducted in the United States, have revealed that survival rate is nearly twice as high as the results of the present study (75% versus 36.2%).

In general, and according to the results of this study, the survival rate of 1, 5 and 10 years for prostate cancer among Asian countries is lower than normal worldwide. Also, countries with higher HDI have higher survival values. These values are higher for Asian people outside of Asia.

In this study, we performed meta-regression for three variables including publication year, HDI level and sample size. The reason for the meta-regression for these three variables was access to data through the included studies. The meta-regression result shows a significant relationship between published year of the studies with five-year survival rate, HDI with one- and five-year survival rate, and sample size with five-year survival rate. The reason for the increase in survival rate over time can be due to introducing new treatments and interventions. Higher HDI can also lead to better access to new treatments and interventions, and this can increase survival rate of patient with prostate cancer.

## Study Limitations

There are certain limitations in systematic review studies, most notably in the absence of access to some of the information that attempts were made to contact the authors of the study to resolve the problem, which in several cases did not receive an adequate response. One of the main limitations of our study was the failure to report sample size and the inability to calculate the confidence interval for survival, which did not allow the study to include the meta-analysis stage. Other limitations included a survival report of less than one-year (6 and 9 months), which, given the low level of these, had no significant effect on our results. Ultimately, due to the lack of studies reporting 10-year survival, the correct estimate of survival requires more robust studies.

## Recommendations for future research

According to the results of this study, estimating the survival rate of prostate cancer requires more extensive studies at the level of other Asian countries, especially in the West and Central Asia, as most studies in this study were conducted in South and Southeast of Asia, and estimates are somewhat incorrect. Another suggestion could be a study of the survival of prostate cancer in patients who metastasized, which was not our study goal, and is an important issue in clinical decision making and the continuation of treatment.

## Conclusion

According to the results of our study, the prostate cancer survival rate in Asian countries is relatively lower than in Europe and North America, which may be due to less access to diagnostic facilities and higher age at recognition of disease than to advanced countries. Another result of our study was the higher survival rate of prostate cancer in countries with very high HDI (such as South Korea and Japan), with similar survival rates as those of advanced countries such as Europe and North America.

## Additional File

The additional file for this article can be found as follows:

10.5334/aogh.2607.s1 Appendix 1. Quality assessment of included studies.
