# Supplementary material for: Survival Rate of Prostate Cancer in Asian Countries: A Systematic Review and Meta-Analysis
Source: Ann Glob Health. 2020 Jan 2;86(1):2. doi: 10.5334/aogh.2607 (PMC6966336; doi:10.5334/aogh.2607)
Supplement: Appendix 1. — Quality assessment of included studies. [file agh-86-1-2607-s1.pdf]

Appendix 1. Quality assessment of included studies

**Author (year)**	**Selection**	**Comparability**	**Outcome**	**Total**	**Quality**
Esteban (1998)	2	1	2	5	Fair
Fan Jin (1998)	2	1	2	5	Fair
Sato (2002)	3	1	2	6	Good
Tsukuma (2006)	3	1	3	7	Good
Jung (2007)	4	1	3	8	Good
Lim (2009)	4	1	3	8	Good
Chia (2010)	4	1	3	8	Good
Chen (2011)	4	1	3	8	Good
Jayalekshmi (2011)	3	1	3	7	Good
Jung (2011)	4	1	3	8	Good
Law (2011)	4	1	3	8	Good
Martin (2011)	3	1	3	7	Good
Matsuda (2011)	3	1	3	8	Good
Sankaranarayanan (2011)	3	1	2	6	Good
Sankaranarayanan (2011)	3	1	2	6	Good
Sriplung (2011)	3	1	3	7	Good
Sumitsawan (2011)	3	1	3	7	Good
Xiang (2011)	4	1	3	8	Good
Xishan (2011)	4	1	3	8	Good
Yeole (2011)	4	1	3	8	Good
Chang (2012)	3	1	3	7	Good
Jung (2012)	4	1	3	8	Good
Balasubramaniam (2013	3	1	3	7	Good
Ito (2013)	4	1	3	8	Good
Jung (2013)	4	1	3	8	Good
Ito (2014)	4	1	3	8	Good

Jung (2014)	4	1	3	8	Good
Takiar (2014)	4	1	3	8	Good
Jung (2015)	4	1	3	8	Good
Maringe (2015)	4	1	3	8	Good
Chang-Mo Oh (2016)	4	1	3	8	Good
Phanphaisarn (2016)	3	1	3	7	Good
Jung (2017)	4	1	3	8	Good
Chen (2018)	3	1	3	7	Good
Chien (2018)	4	1	3	8	Good
Zeng (2018)	4	1	3	8	Good
